# MCT2 Expression and Lactate Influx in Anorexigenic and Orexigenic Neurons of the Arcuate Nucleus

**DOI:** 10.1371/journal.pone.0062532

**Published:** 2013-04-26

**Authors:** Christian Cortes-Campos, Roberto Elizondo, Claudio Carril, Fernando Martínez, Katica Boric, Francisco Nualart, Maria Angeles Garcia-Robles

**Affiliations:** 1 Laboratorio de Biología Celular, Departamento de Biología Celular, Universidad de Concepción, Concepción, Chile; 2 Laboratorio de Neurobiología y Células Madre, Centro de Microscopía Avanzada CMA BIO BIO, Universidad de Concepción, Concepción, Chile; Peking University, China

## Abstract

Hypothalamic neurons of the arcuate nucleus control food intake, releasing orexigenic and anorexigenic neuropeptides in response to changes in glucose concentration. Several studies have suggested that the glucosensing mechanism is governed by a metabolic interaction between neurons and glial cells via lactate flux through monocarboxylate transporters (MCTs). Hypothalamic glial cells (tanycytes) release lactate through MCT1 and MCT4; however, similar analyses in neuroendocrine neurons have yet to be undertaken. Using primary rat hypothalamic cell cultures and fluorimetric assays, lactate incorporation was detected. Furthermore, the expression and function of MCT2 was demonstrated in the hypothalamic neuronal cell line, GT1-7, using kinetic and inhibition assays. Moreover, MCT2 expression and localization in the Sprague Dawley rat hypothalamus was analyzed using RT-PCR, *in situ* hybridization and Western blot analyses. Confocal immunohistochemistry analyses revealed MCT2 localization in neuronal but not glial cells. Moreover, MCT2 was localized to ∼90% of orexigenic and ∼60% of anorexigenic neurons as determined by immunolocalization analysis of AgRP and POMC with MCT2-positives neurons. Thus, MCT2 distribution coupled with lactate uptake by hypothalamic neurons suggests that hypothalamic neurons control food intake using lactate to reflect changes in glucose levels.

## Introduction

Neurons within the arcuate nucleus (AN) of the hypothalamus are capable of responding to changes in glucose and lactate concentrations [1], [2]. These neurons couple blood glucose fluctuations with a complex network of neurochemical and neurophysiologic responses that control energy expenditure and feeding behavior [3]. The hypothalamic melanocortin pathway plays a critical role, as it is directly regulated by hormones and nutrients [4]. The melanocortin pathway is formed from neuronal populations localized in the AN with antagonistic actions. Orexigenic neurons that synthesize neuropeptide Y (NPY) and agouti-related peptide (AgRP) increase food intake and reduce energy expenditure. Contrary effects were observed when anorexigenic neurons, including proopiomelanocortin (POMC) neurons, are stimulated [3]. On the other hand one population within the hypothalamus, glucose-exited neurons (GE), increases its firing rate in response to elevated glucose levels. Alternative, neurons that reduced their firing rate in response to increased glucose levels are known as glucose-inhibited neurons (GI) [5], [6]. A high percentage of GI neurons are immunoreactive for NPY [7]. In contrast, it has been proposed that POMC neurons could be correspond to GE neurons [8], [9].

AN neurons are not in direct contact with blood or cerebrospinal fluid, and therefore, cannot directly detect and respond to changes in glucose concentration [5], [10], [11], [12], [13]. Previous studies have proposed that the hypothalamic glial cells (i.e., tanycytes) release lactate to neighboring neurons through monocarboxylate transporters (MCT) in response to high glucose, resulting in neuronal activation [11], [12], [14], [15], [16], [17]. Furthermore, dynamic bioluminescence imaging, which records cytosolic ATP ([ATP]_c_) in real-time, revealed no significant changes in [ATP]_c_ (<2%) in response to high glucose (15 mM) in hypothalamic neurons, contrary to expected results. However, these neurons responded to lactate with a significant increase in [ATP]_c_ [14], suggesting that lactate but not glucose induces a neuronal response in high glucose conditions. The participation of lactate in the glucosensing mechanism and feeding behavior is supported by a study by Lam et al. [18] in which hypothalamic injection of lactate mimicked the effects of hypothalamic glucose administration. Moreover, GE neurons located in the VMH respond to lactate (15 mM) [2].

Tanycytes release lactate through MCTs, and β1 ventral tanycytes (β1v), which contact an orexigenic area of AN, express MCT1 [10]. MCT4 distribution in the processes of β1 dorsal tanycytes (β1d) contacting the anorexigenic zone of AN was also observed [11]. Moreover, the selective distribution of MCTs between neurons and glial cells suggests that lactate plays a key role in brain energy metabolism, and that MCT2 is essential for neuronal lactate uptake [19], [20], [21], [22], [23]. However, the expression of MCTs in hypothalamic AN neurons in normal animals has not been analyzed to date.

In this study, the expression and function of MCT2 in hypothalamic neurons was analyzed. Using primary hypothalamic cell cultures and the hypothalamic neuronal cell line, GT1-7, incorporation of lactate was observed through MCT2. Moreover, RT-PCR, *in situ* hybridization, Western blot, and immunohistochemistry analyses demonstrated MCT2 localization in orexigenic and anorexigenic neurons. The distribution of MCTs in the hypothalamus suggests that neuronal and glial cells interact through lactate to regulate the activity of neurons that control food intake.

## Materials and Methods

### Ethics Statement

All animals were handled in strict accordance with the Animal Welfare Assurance (permit number 2010101A), and all animal work was approved by the appropriate Ethics and Animal Care and Use Committee of the Universidad de Concepcion, Chile. Male adult Sprague-Dawley rats between 200–300 g and mouse C57BL/6N were used for the experiments. Animals were kept in a 12-h light/dark cycle with food and water *ad libitum*.

### Primary Cell Culture

A mixed primary culture of hypothalamic neurons and glia was prepared from Sprague-Dawley rats at 19 days of gestation and maintained in culture at 37°C and 5% CO_2_. Briefly, the hypothalamic region of the brain was microdissected at 4°C in buffer containing 10 mM HEPES (pH 5 7.4; 340 mOsm/L). Subsequently, the tissue was subjected to enzymatic desegregation for 10 min at 37°C in 0.25% trypsin (Invitrogen, Rockville, MD, USA) and EDTA 0.20% (Sigma-Aldrich, St. Louis, MO, USA). After the tissue was then transferred to MEM (Invitrogen) with 10% (v/v) fetal bovine serum (FBS) (Thermo Fisher Scientific Inc., Waltham, MA, USA) and 2 mg/mL DNAse I (Sigma-Aldrich), mechanical disaggregation was performed. The cells (∼5×10^5^ cells/cm^2^) were plated on coverslips 18 mm in diameter that were coated with 0.2 mg/mL poly-L-lysine (Sigma-Aldrich) in 12-well plates (Corning Costar, NY, USA). After 30 min, Neurobasal medium supplemented with B27, 2 mM glutamine, 100 U/mL penicillin, 100 mg/mL streptomycin, and 2.5 mg/mL Fungizone (all Invitrogen) was added. After 48 h, the cultures were treated with 15 µg Ara-C (Sigma-Aldrich) and maintained in culture for 7 days until subsequent immunocytochemistry and fluorimetric analyses.

### Immortalized Cell Culture

The mouse hypothalamic tumor cell line, GT1-7 [24] (kindly provided by Dr. P. Mellow) was maintained at 37°C and 5% CO_2_ on Petri dishes (Corning Costar) in high glucose DMEM supplemented with 10% FCS, 3.75 mM glutamine, and 2.5 µg/mL Fungizone (all Invitrogen), according to [24]. The dishes with the highest density of confluent cells were expanded (1∶5) and used for immunocytochemistry, lactate uptake, and immunoblot experiments.

### Immunocytochemistry

Cells (5×10^5^ cells/cm^2^) were grown on 0.2 mg/mL poly-L-lysine-coated glass cover slides (Sigma-Aldrich) in 24-well plates and fixed with 4% paraformaldehyde in PBS for 30 min. The immunocytochemistry was performed as previously described [11], using the following primary antibodies: mouse anti-MAP2 (1∶200, Millipore, Temecula, CA, USA), chicken anti-Vimentin (1∶200, Millipore), mouse anti-neurofilaments (1∶1, Hibrydoma Data Bank), mouse anti-N-CAM (1∶1, Hibrydoma Data Bank), rabbit anti-POMC (1∶200, Phoenix Pharmaceuticals, Burlingame, CA, USA), goat anti-CART (1∶200, R&D Systems, MN, USA), goat anti-AgRP (1∶200, R&D Systems) and rabbit anti-NPY (Millipore), chicken anti-MCT1 (1∶100, Millipore), chicken anti-MCT2 (1∶20, Millipore), and rabbit anti-MCT4 (1∶100, Millipore). The slides were analyzed using confocal laser microscopy (D-Eclipse C1 Nikon, Tokyo, Japan).

### Fluorimetric L-Lactate Analyses

Changes in intracellular pH (pHi) in hypothalamic neurons were visualized using fluorescence measurements of intracellular 2′,7′-bis-(2-carboxyethyl)-5-(and-6)-carboxyfluorescein acetoxymethyl ester (BCECF-AM). Cells were incubated in cell culture medium with 5 µM BCECF-AM (Invitrogen) for 30 min at 37°C in a 95% O_2_-5% CO_2_ atmosphere. After loading the probe, the cells were washed three times with HBSS/sNa-HCO_3_ buffer (125 mM N-methyl glucamide, 5 mM KCl, 1 mM CaCl_2_, 1.2 mM MgSO_4_, 2 mM KH_2_PO_4_, 32 mM HEPES, 10 mM glucose, pH 7.4 and 320 mOsm). To inhibit the main H^+^ transport system in neurons, dicyclohexylcarboxiimide, a nonspecific H^+^-ATPase inhibitor, was added to the cells 20 min before the pHi measurements [25]. The excitation fluorescence ratio of 495/440 nm was determined using an emission wavelength of 535 nm in a Ti microscope (Nikon) conditioned with search filters and D495/10X D440/10X excitation, emission D535/25m and dichroic 515dcxr (Chroma Technology Corp., Bellows Falls, VT, USA) and a CoolSNAP HQ2 camera (Photometrics, Tucson, AZ, USA). After a basal fluorescence ratio was obtained 10, 20 and 50 mM of L-lactate were added to the cell suspension, and the fluorescence ratio was recorded until it reached a new steady-state level. All experiments were performed in triplicate, and results represent an average of at least two separate experiments.

### L-Lactate ^14^C Uptake Analysis

For lactate uptake assays, GT1-7 cells (5×10^5^ cells/well) were seeded in 0.2 mg/mL poly-L-lysine coated 12-well plates (Sigma-Aldrich) and grown for 24 h up to 95% confluence. Cultures were carefully selected under the microscope to ensure that only plates showing uniformly growing cells were used. Lactate uptake analysis was performed as previously described [11] using 1–2 µCi of L-[^14^C(U)] lactic acid sodium salt (>100 mCi [3.70 GBq]/mmol; PerkinElmer-NEN, Boston, MA, USA). In L-Lactate inhibition experiments, cells were pre-incubated with 5 mM alpha-cyano-4-hydroxycinnamate (4-CIN, Sigma-Aldrich), 3 mM p-chloromercuribenzene sulfonate (pCMBS, Sigma-Aldrich), 1mM floretin (Sigma-Aldrich), or 1 mM di-isothiocyanostilbene disulfonate (DIDS, Sigma-Aldrich) for 15 min at 37°C (incubation buffer). For competitive analyses, cells were co-incubated with 10 mM L-pyruvate at 4°C. All inhibition experiments were carried out under or at initial velocity conditions to discriminate between L-lactate transport and metabolism.

### *In situ* Hybridization

Using sense 5′-GTC TCA TCT CCG AAT CAG TGT TCA G-3′ and antisense 5-CCC GTT ACT CAG TGT TTG CGT G-3′ primers, a PCR product of 469 bp was obtained from the hypothalamus and subcloned into pCR®-4-Blunt-TOPO® (Invitrogen) to generate sense and antisense digoxigenin-labeled riboprobes. RNA probes were labeled with digoxigenin-UTP by *in vitro* transcription with T3 or T7 RNA polymerase following the manufacturer's procedure (Invitrogen). *In situ* hybridization was performed on rat frontal brain sections of 7 µm mounted on poly-L-lysine-coated glass slides [26]. The sections were baked at 60°C for 1 h, deparaffinized in xylene, and rehydrated in graded ethanol. Following incubation with 1 µg/ml proteinase K (Sigma) in PBS (pH 7.4) for 5 min at 37°C, the tissue sections were fixed with 4% paraformaldehyde at 4°C for 5 min, washed in cold PBS, and then acetylated with 0.1 M triethanolamine-HCl (pH 8.0) and 0.25% acetic anhydride at room temperature for 10 min. After a brief wash with PBS, the sections were incubated in pre-hybridization solution (Novagen) at 37°C for 30 min, and then 25 µl of hybridization mix (50% formamide, 0.6 M NaCl, 10 mM Tris-HCl [pH 7.5], 1 mM ethylenediaminetetraacetic acid [EDTA], 1× Denhart’s solution, 10 mM DL-dithiothreitol [DTT], 500 µg/mL yeast tRNA, 50 µg/ml heparin, 500 µg/ml DNA carrier, and 1∶25 dilution of riboprobe) were added to each slide. The slides were covered with glass coverslips and placed in a humidified chamber at 42°C overnight. After removal of the coverslips, the slides were rinsed in 4× saline-sodium citrate (SSC) and washed twice for 15 min at 42°C. The slides were washed twice at 37°C for 15 min each in 2× SSC, 1× SSC and 0.3× SSC. Visualization of digoxigenin was performed by incubation with a monoclonal antibody coupled to alkaline phosphatase (anti-digoxigenin alkaline phosphatase Fab fragments diluted 1∶500; Roche Applied Science) at room temperature for 2 h. Nitroblue tetrazolium chloride and 5-bromo-4-chloro-3-indolyl-phosphate (Roche Applied Science) were used as substrates for the alkaline phosphatase. Controls included use of the sense riboprobe and omission of the probe.

### Intracerebral Ventricular (i.c.v) Injection of Colchicine

Rats were anesthetized with an intraperitoneal injection mix of ketamine-xilazine (90 mg/Kg-10 mg/Kg). Using a cannula (28 gauge), 10 µL of 10 mg/mL colchicine (Sigma-Aldrich) was injected into the third ventricle (AP −3.14 mm, ML 0.0 mm, DV 9.2 mm). After 20 h, rat brains were fixed for vascular perfusion using 4% PFA and processed for cryosectioning into 30-µm sections. The samples were used for subsequent immunohistochemical detection of neuropeptides.

### Immunohistochemistry

Immunohistochemical analyses was performed as previously described [10] using the following antibodies and dilutions: rabbit anti-GLUT1 (1∶100, Alpha Diagnostic International, INC., San Antonio, TX, USA), rabbit anti-glial fibrillary acidic protein (GFAP; 1∶200, Dako, Campintene, CA, USA), mouse anti-vimentin (1∶200, Dako), chicken anti-MCT2 (1∶20, Millipore), rabbit anti-POMC (1∶200, Phoenix Pharmaceuticals), goat anti-CART (1∶200, R&D Systems), goat anti-AgRP (1∶200, R&D Systems), and rabbit anti-NPY (Millipore).

### Image Analyses

To analyze MCT2/vimentin, MCT2/GLUT1, MCT2/GFAP, vimentin/GLUT1 and vimentin/GFAP co-localization within various regions of interest in the hypothalamic region, NIS-Elements software (Nikon, Nikon Instruments INC), was used, and Pearson’s coefficient (Rr) values were calculated. This coefficient measures the overlapping level between the pixels of two fluorescent channels, ranging from +1 to −1 (0–100% co-localization) [27]. The statistical analysis was performed comparing the Rr calculated between the glial markers (control) with the Rr calculated between the control and the protein of interest.

Quantification of POMC- and AgRP-positive neurons to MCT2 was performed using ImageJ® software (National Institute of Health, Bethesda, Maryland, USA). Each image was analyzed as follows. A Gaussian Blur filter of 1 pixel size was applied to the POMC and AgRP channels. Using a threshold of 75, the significant intensity areas over 190 pixels were selected. Subsequently, only the areas with an average intensity greater than 130 were chosen and selected as POMC or AgRP cells. After that, only those areas whose average MCT2 channel intensity was over 1 and whose minimum MCT2 intensity was less than 78 were selected for analysis, excluding the cells out of the region of interest. The MCT2 intensity inside every POMC and AgRP cell was calculated and only those with a sum over 12000 were chosen as MCT2-positive cells. For cells that were within clusters, the number of cells forming each cluster was estimated by comparing the cluster size and the average object size. With this data, the number of POMC/MCT2- positive, AgRP/MCT2-positive cells were analyzed.

### Reverse Transcription-polymerase Chain Reaction (RT-PCR)

The brain of each rat and brain of mouse were removed, and the hypothalamic area or medulla renal was isolated and further dissected. Total RNA from hypothalamus, control tissues, or cell cultures were isolated using Trizol (Invitrogen) and treated with DNase I (Fermentas International INC, Burlington, Ontario, Canada) to remove genomic DNA contamination. The RT-PCR was performed according to the manufacturer’s protocol using 2 µg RNA (Fermentas International INC). Parallel reactions were performed in the absence of reverse transcriptase to control for the presence of contaminant genomic DNA. The PCR reaction was performed using 1 µL cDNA and the following sets of primers: MCT1, sense 5-GGG AAG GTG GAA AAA CTC AA-3′ and antisense 5′-ACA CTC CAT TCG CAA CAA CA-3′ (expected product of 400 bp, in rat and mouse); MCT2, sense 5′- CAG GAG GTC CCA TCA GTA GT -3′and antisense 5′- ACT TTT AGA CTT CGC AGC AC -3′ (expected product of 416 bp, in rat); MCT2, sense 5′ TCA GCT CTG CAA TGA TGT TT - 3′and antisense 5′- AGG GAG GAT TGT GTG CGT TT -3′ (expected product of 476 bp, in mouse); MCT4, sense 5- TGC GGC CCT ACT CTG TCT AC -3′ and antisense 5′- TCT TCC CGA TGC AGA AGA AG -3′ (expected product of 369 bp, in mouse); and β-actin, sense 5′-GCT GCT CGT CGA CAA CGG CTC-3′ and antisense 5′- CAA ACA TGA TCT GGG TCA TCT TCT C-3′ (expected product of 353 bp). Each reaction mixture was incubated at 95°C for 5 min followed by 35 cycles of 30 s at 95°C, 30 s at 55°C, and 30 s at 72°C and a final extension of 7 min at 72°C. PCR products were separated by 1.2% agarose gel electrophoresis and visualized by staining with ethidium bromide.

### Immunoblotting

Western blot analysis was performed as previously described [11], using total protein extracts obtained from rat hypothalamus, cerebral cortex, and GT1-7 cell cultures and the following antibodies: chicken anti-MCT1 (1∶4000, Millipore), chicken anti-MCT2 (1∶1000, Millipore), and peroxidase-labeled rabbit anti-chicken IgY (1∶1000; Jackson Immuno Research). Negative controls were performed by incubating the membrane with a pre-absorbed antibody (anti-MCT1 1:100 or anti-MCT2 1:100 with 100 µg/mL inductor peptide incubated at 4°C overnight, Aves Lab).

### Statistical Analysis

All values are expressed as the means ± SEM. Statistical comparisons between groups were performed using one-way ANOVA followed by Fisher’s test among more than three groups or the unpaired Student’s t-test for two groups. All analyses were performed using GraphPad Prism 4.0 Software (GraphPad Software Inc., San Diego CA).

## Results

### Functional Characterization of MCT2 in Primary Hypothalamic Cultures and in the GT1-7 Cell Line

**Figure 1:**
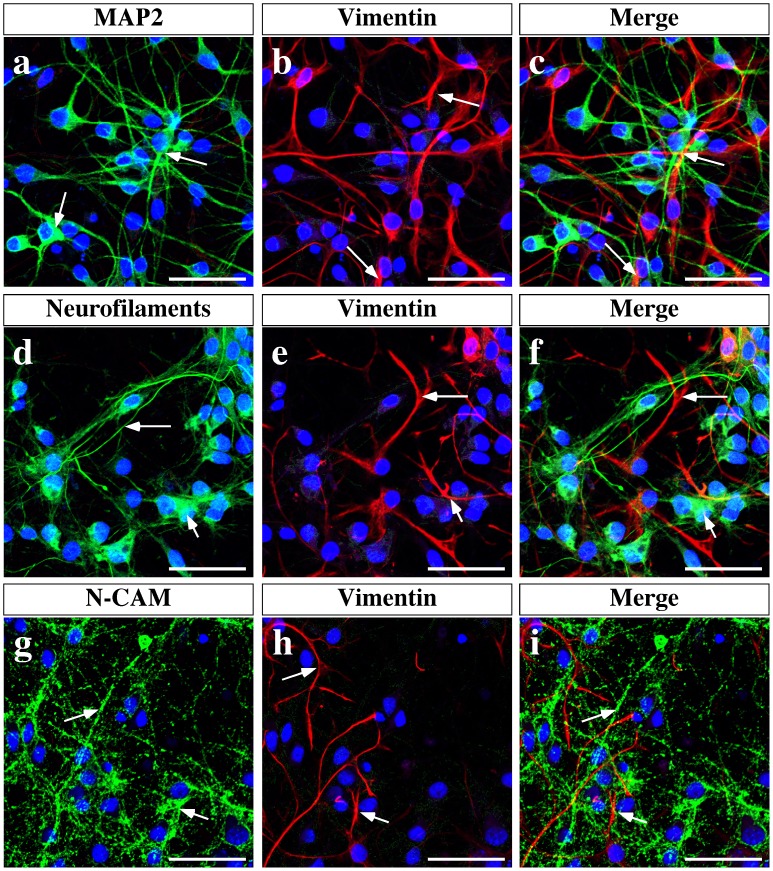
Immunocharacterization of mixed primary cultures enriched in hypothalamic neurons. Neurons obtained from rat hypothalamus at 19 days of gestation were cultured for 2 weeks. (a–i) Representative confocal images depicting MAP2 (a, green), Vimentin (b, e, and h, red), Neurofilaments (d, green), and N-CAM (g, green). Nuclei were stained with TOPRO-3 (blue). Triple labeling shows that the cultures are enriched in neurons (c, f and i). Scale bar: 80 µm.

**Figure 2:**
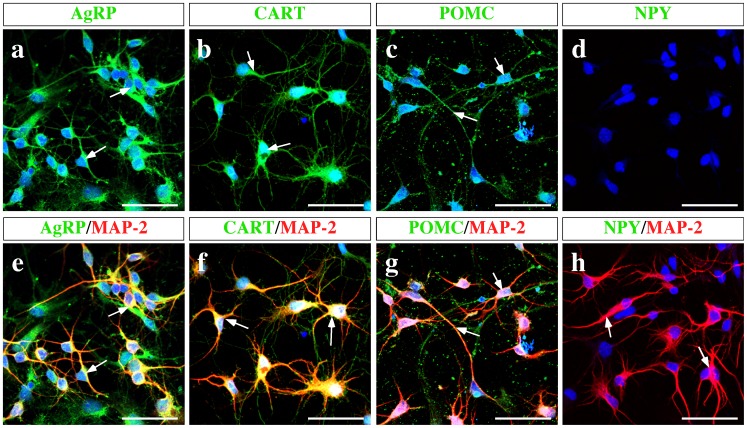
Orexigenic and anorexigenic neuropeptides are expressed in hypothalamic primary cell culture. (a–d) Representative confocal images of AgRP (a, green), CART (b, green), POMC (c, green), and NPY (d, green) cells. The neuronal identity was confirmed using a MAP2 antibody (e–h). Scale bar: 80 µm.

Tanycytes within the AN release lactate through MCT1 and MCT4 in response to glucose [11]. Therefore, the uptake of lactate by hypothalamic neurons was evaluated; MCT expression in these cells was also assessed. Mixed primary cultures enriched in hypothalamic neurons were assessed for neuronal markers, such as MAP-2 (Fig. 1a, arrows), neurofilaments (Fig. 1d, arrows), and N-CAM (Fig. 1g, arrows), and the tanycyte marker, vimentin (Fig. 1b, e and h, arrows), by immunocytochemistry. There were two distinguishable cell populations that did not co-express these markers, neurons and tanycytes (Fig. 1c, f and i, arrows). The co-expression of the neuropeptides, AgRP, CART, POMC and NPY, with the neuronal marker, MAP-2, was also analyzed (Fig. 2a–h); *in vitro* hypothalamic neurons expressed the orexigenic neuropeptides, AgRP (Fig. 2a and e, arrows), the anorexigenic neuropeptides CART (Fig. 2b and f, arrows), and POMC (Fig. 2c and g, arrows). However, NPY was not detected (Fig. 2d and h, arrows) in the cell culture.

**Figure 3:**
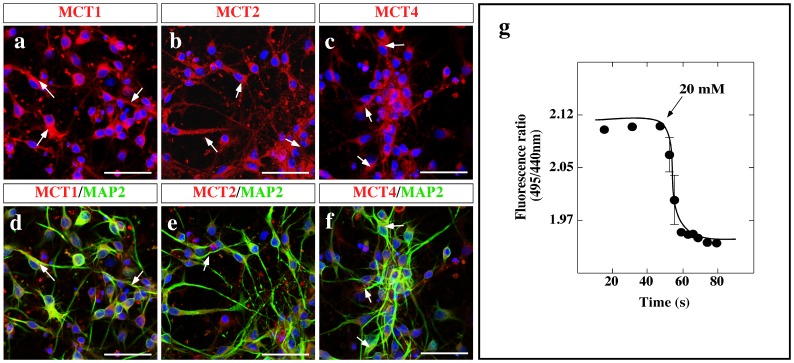
MCT analysis in hypothalamic primary cell culture. (a–f) Representative confocal images of MCT1 (a, red), MCT2 (b, red), and MCT4 (c, red) neurons. Co-distribution of MCT1, MCT2, and MCT4 with MAP2 (d–f, green). Nuclei were stained with TOPRO-3 (blue). Scale bar: 80 µm. (g) Fluorescence ratio of neurons as a function of time. The cells were incubated for 10 min with dicyclohexylcarbodiimide (DCCD; 50 mM) to inhibit the V-type H^+^-ATPase in these cells. At the indicated times, 20 mM L-lactate was added at 18°C.

After confirming the presence of orexigenic and anorexigenic neurons, the expression of MCTs and lactate uptake was assessed. Immunocytochemistry analyses revealed that cultured neurons expressed MCT1 (Fig. 3a, arrows), MCT2 (Fig. 3b, arrows), and MCT4 (Fig. 3c, arrows), which were co-distributed with MAP-2 in this culture (Fig. 3d–f, arrows).

Lactate incorporation via MCTs was also analyzed in these mixed hypothalamic primary cultures using fluorimetric assays. Since MCTs are monocarboxylate/H^+^ symporters, the *in vivo* uptake of lactate was undertaken in the presence of H^+^-ATPase inhibitors and in the absence of bicarbonate. The response of the neurons to the addition of 20 mM L-lactate was detected as a decrease in the ratio of fluorescence that stabilized a few seconds after lactate was added (Fig. 3g), indicating that hypothalamic neurons incorporate lactate. However, using concentrations below 20 mM of L-lactate, reproducible changes in the fluorescence ratio were not observed (data not shown); therefore, this technique did not permit functional characterization of the MCT isoform present in this culture. To resolve this problem, MCT expression and lactate uptake was subsequently analyzed in the hypothalamic neuron cell line, GT1-7 [24], [28].

**Figure 4:**
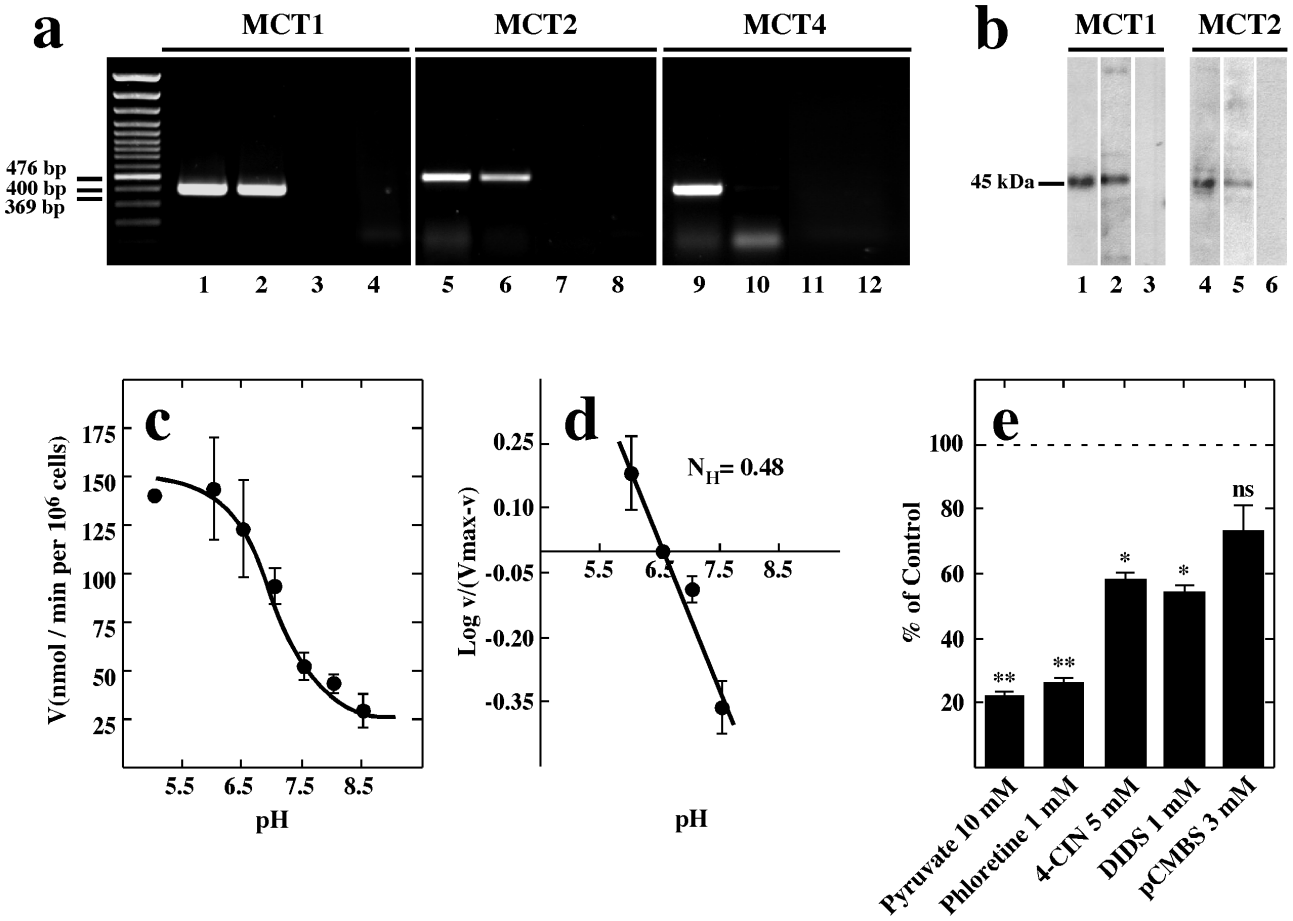
MCTs are expressed in the GT1-7 cell line. (a) RT-PCR and (b) immunoblot analyses of MCT1, MCT2 and MCT4 mRNA and protein expression, respectively. RNA isolated from mouse hypothalamus (lanes 1, 5 and 9) and GT1-7 cultures (lanes 2, 6 and 10). RT(−) of GT1-7 culture (lanes 3, 7 and 11). Total protein extracted from mouse hypothalamus (lanes 1 and 4) and GT1-7 (lanes 2–3 and 5–6). (c) Dependence of lactate uptake on pH at 0.1 mM L-lactate and 20°C. (d) Hill plot to analyze the dependence of lactate uptake on pH. (e) Analysis of lactate transport in the presence of various inhibitors co-incubated for 1 min with 0.1 mM lactate at 20°C and pH 7.0.

The expression of MCT1, MCT2 and MCT4 in GT1-7 cells was analyzed using RT-PCR with mouse-specific primers and conditions optimized using RNA from mouse hypothalamus. The amplified cDNA bands were 400 and 476 and 369 bp, which are the expected sizes for MCT1, MCT2 and MCT4, respectively (Fig. 4a, lanes 1, 5 and 9). GT1-7 cells expressed MCT1 and MCT2 but not MCT4 (Fig. 4a, lanes 2, 6 and 10). No amplification product was observed in samples without reverse transcriptase (Fig. 4a, lane 3, 7 and 11) or water (Fig. 4a, lane 4, 8 and 12), indicating the absence of genomic DNA or contamination in the PCR reaction. MCT1 and MCT2 expression was also demonstrated using immunoblot analysis of proteins isolated from rat hypothalamus (positive control) (Fig. 4b, lanes 1 and 4) and GT1-7 cells (Fig. 4b, lanes 2 and 5). Analysis of hypothalamus protein extract using anti-MCT preabsorbed with inductor peptides showed an absence of bands (Fig. 4b, lanes 3 and 6). Therefore, RT-PCR and Western blots analyses confirmed the expression of MCT1 an MCT2 in GT1-7 cells.

**Figure 5:**
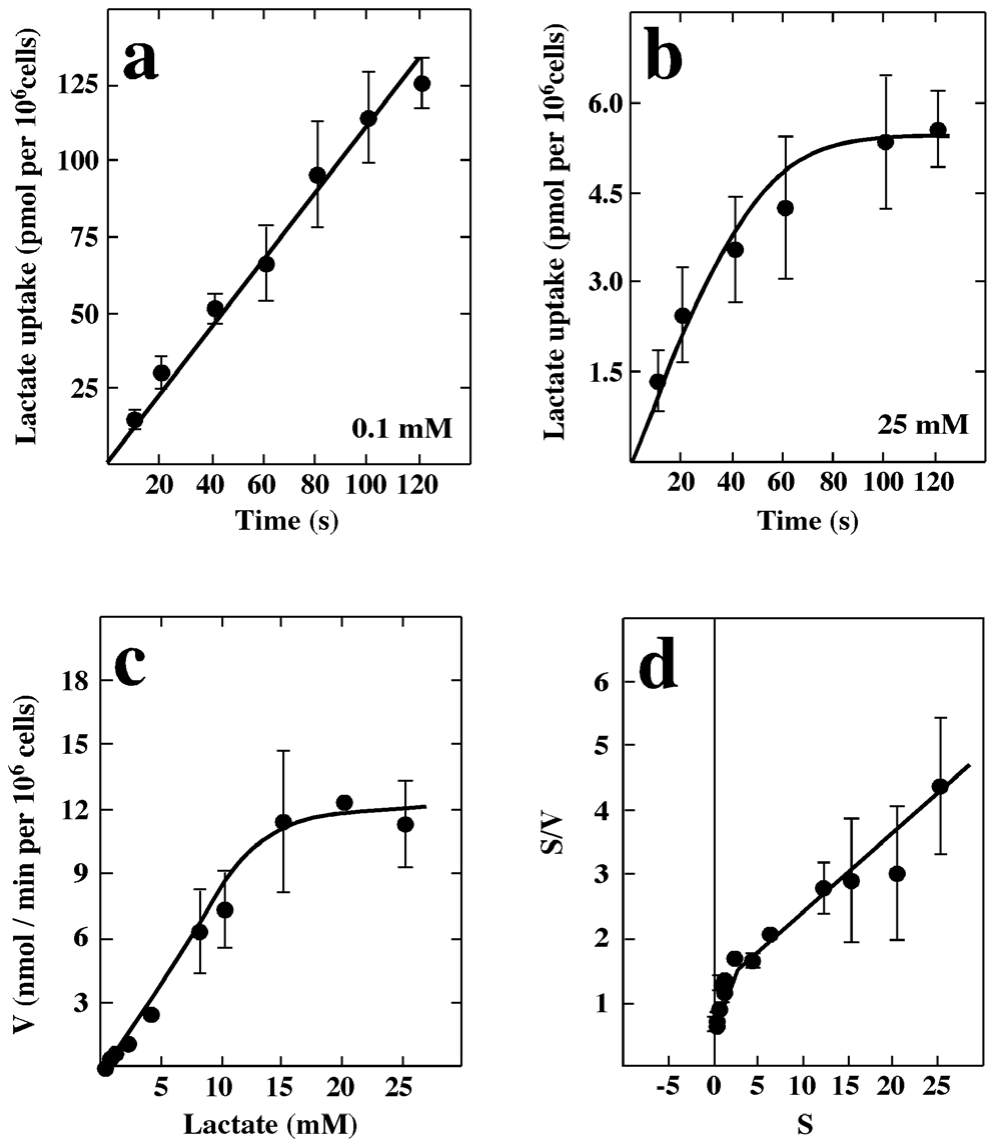
Functional characterization of MCT in the GT1-7 cell line. (a) Time course of 0.1 mM and (b) 25 mM L-lactate at 4°C and pH 7.0. (c) Kinetic parameters of L-lactate transport in GT1-7 cells after 30 s at 4°C, and pH 7.0. (d) Hanes–Woolf plot of MCT1: (Km, 2.2±0.4 mM; V_max_, 3 nmol/min per 10^6^ cells) and MCT2: (Km, 0.3±0.1 mM; V_max_, 1 nmol/min per 10^6^ cells). Results represent the mean ± SD of three independent experiments. **p<0.001, one tailed t-test.

Transport assays revealed a dependence on pH for lactate uptake in GT1-7 cells. At a substrate concentration of 0.1 mM, lactate uptake at pH 5.0 was more than five times faster than that observed at pH 8.5 when the assays were performed at 20°C (Fig. 4c). The proton effect was found to be of a non-cooperative nature, which was corroborated by a Hill plot that yielded a straight line with a slope (Hill coefficient) of 0.48 (Fig. 4d). To determine whether other monocarboxylates might also serve as substrates for the lactate transporter in GT1-7 cells, competition experiments were performed. At an extracellular concentration of 0.1 mM lactate (20°C, pH 7.0), transport was strongly inhibited by 10 mM pyruvate (Fig. 4e). Incubation with MCT inhibitors, including 1 mM phloretin, 5 mM 4-CIN and 1 mM DIDS, decreased lactate uptake up to 70%, 40% and 50% respectively. However, 3 mM pCMBS did not significantly inhibit lactate uptake (Fig. 4e). The uptake of 0.1 and 25 mM lactate at 4°C was almost linearly correlated up to 60 s (Fig. 5a and b). Using these optimized conditions, the basic transport parameters for lactate uptake in GT1-7 cell culture were determined. Dose-response studies performed up to 30 s revealed that lactate transport was saturated at concentrations above 15 mM (Fig. 5c). Data analysis suggested the presence of two kinetic components for lactate transport. When the data was transformed and plotted according to Hanes–Woolf, this observation was confirmed (Fig. 5d). Transformation showed apparent Km values of 0.5 mM and 6 mM and V_max_ values of 1.5 (nmol/min per 10^6^ cells) and 15 (nmol/min per 10^6^ cells), corresponding to MCT2 and MCT1 respectively.

### MCT2 is Expressed in the Hypothalamus

**Figure 6:**
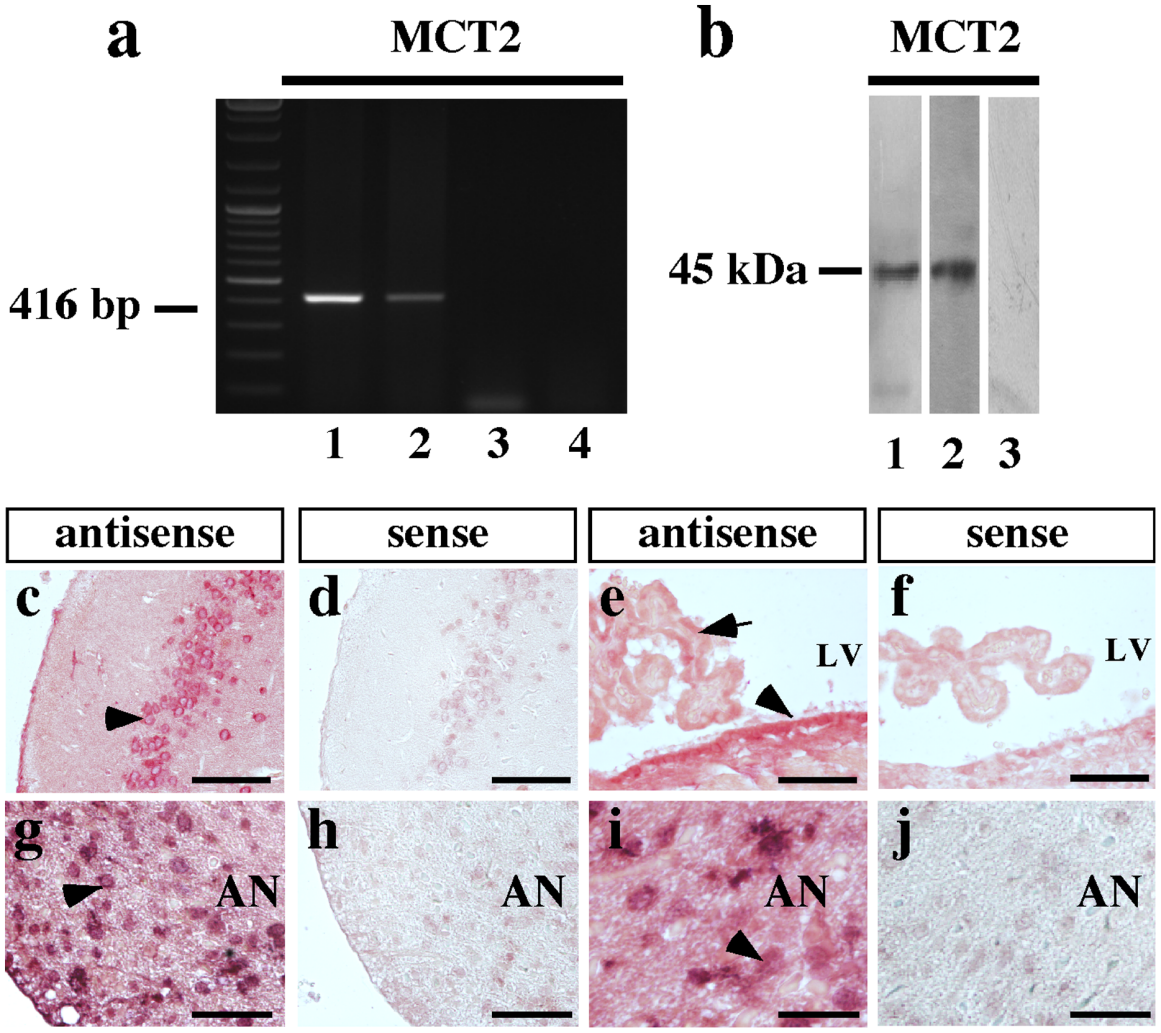
MCT2 expression in adult rat hypothalamus. (a) RT-PCR analysis of RNA isolated from rat cerebral cortex (lane 1) and rat hypothalamus (lanes 2–3). RT(−) (lane 3) and water in the PCR reaction (lane 4). (b) Immunoblot analysis of total protein extracted from rat cerebral cortex (lane 1) and rat hypothalamus (lane 2). Negative control was performed with primary antibodies pre-adsorbed with inductor peptide (lane 3). (c–h) Neuronal MCT2 mRNA detection by *in situ* hybridization. Frontal section of rat brain probed with a MCT2 antisense riboprobe. A representative panoramic image showing high hybridization signal in basal hypothalamus (c), in neurons of entorrinal cortex (d). Ependymal cells of lateral ventricle and some choroidal cells present positive hybridization with antisense MCT2 riboprobe (e). In the hypothalamus, a high hybridization signal was observed in neurons of peri-ventricular (g) and distal (i) arcuate nucleus. Negative reaction was obtained using sense riboprobes in control areas (insets in c–g). AN: arcuate nucleus, LV: lateral ventricle. Scale bar: (c–f) 50 µm; (g–j) 20 µm.

The expression of MCT2 in rat hypothalamus was analyzed by RT-PCR with specific primers (Fig. 6a). The amplified DNA band was approximately 416 bp, which was the expected size for the MCT2 amplification product (Fig. 6a, lanes 2). The PCR conditions were optimized using RNA from the cerebral cortex as a positive control for MCT2 expression [28] (Fig. 6a, lane 1). No amplification product was observed in samples without reverse transcriptase (Fig. 6a, lane 3) or water (Fig. 6a, lane 4). Immunoblot analysis using protein extracts isolated from the cerebral cortex (positive control) and hypothalamus showed MCT2 expression (45 kDa band) in both tissues (Fig. 6b, lanes 1 and 2). Bands were absent in samples incubated with anti-MCT2 preabsorbed with inductor peptides (Fig. 6b, lanes 3). MCT2 mRNA expression was also analyzed using *in situ* hybridization with digoxigenin-labeled cRNA probes specific for MCT2 (Fig. 6c–g). A positive hybridization signal was observed in neurons of the entorhinal cortex (Fig. 6d, arrow head), in the ependymal layer of lateral ventricle (Fig. 6e, arrow head) and in some choroid plexus cells (Fig. 6e, arrow). In the basal hypothalamus (Fig. 6c), the reaction was concentrated in the AN neurons, principally in the peri-ventricular (Fig. 6g, arrow head) and lateral regions of the AN (Fig. 6h, arrows head). Negative hybridization was observed using the sense probe (Fig. 6 inset in c–g).

### MCT2 is Localized in Hypothalamic Neurons

The high specificity of the antibodies used in this study was evaluated in control tissues and several cerebral areas (Fig. S1) [19], [20], [23], [29], [30], [31], [32]. Anti-MCT2 reactivity was localized in the kidney (Fig. S1a) and brain (Fig. S1b–h). Immunohistochemical studies revealed MCT2 reactivity in some collecting ducts of the kidney medulla (Fig. S1a, arrows) and in brain endothelial cells (Fig. S1b, arrows). Co-distribution analyses performed with anti-MCT2 and anti-GLUT1 in the brain cortex showed MCT2 immunoreaction in astrocytes that surrounded GLUT1-positive vessels in several regions of the brain cortex and in the marginal zone (Fig. S1c–e, arrows). Co-distribution was only detected in microvessels (Fig. S1c–d, arrowheads). Intense immunoreaction was detected in ependymal cells of the lateral ventricle (Fig. S1f–g, arrows) and subependymal astrocytes of the dorsal third ventricle (Fig. S1h, arrows).

**Figure 7:**
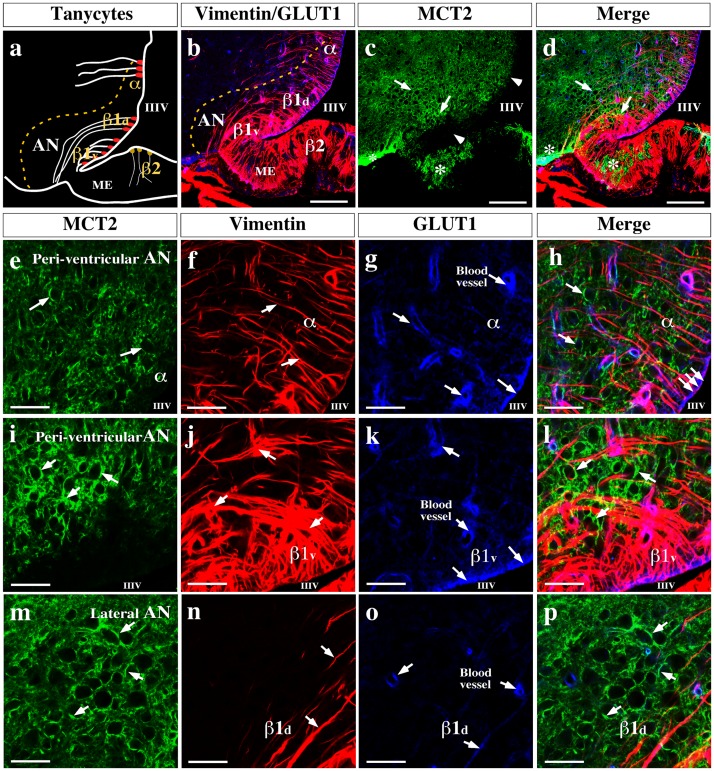
MCT2 is localized in the arcuate nucleus. (a) Schematic representation of the hypothalamic area shown in b–d. (b) Rat frontal brain section using anti-vimentin (red) and anti-GLUT1 (blue) antibodies, markers of glial cells. (c) MCT2 localization. (d) Rat frontal brain section using anti-vimentin (red), anti-GLUT1 (blue), and anti-MCT2 (green) antibodies. (e–h) MCT2 was observed in cellular membranes (e) of dorsal peri-ventricular arcuate nucleus areas; this area was negative for vimentin (f, h) and GLUT1 (g, h). GLUT1 and vimentin are co-distributed in the apical region of α-tanycytes (h, triple-arrows). (i–l) In the ventral peri-ventricular area, which contains β1-tanycytes, MCT2 was expressed in cellular membranes; this area was negative for vimentin (j) and GLUT1 (k). GLUT1 and vimentin are co-distributed in apical region of β1-tanycytes (arrows). (m–p) MCT2 was observed in the cellular membranes of β1-tanycyte processes in the ventral lateral area of the arcuate nucleus, which was negative for vimentin (n, p) and GLUT1(o, p). AN: arcuate nucleus, III–V: third ventricle, ME: median eminence. Scale bar: (b and e) 150 µm; (c–d and f–u) 50 µm.

**Figure 8:**
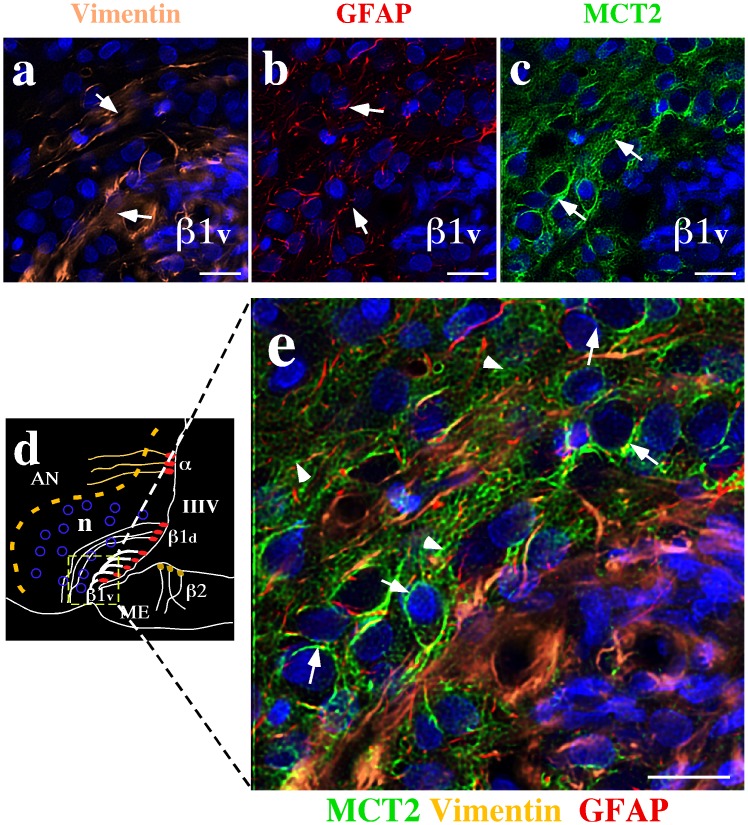
MCT2 is expressed in arcuate nucleus neurons. (a–c) Low magnification analysis of the basal hypothalamic area using anti-vimentin (a, orange), anti-GFAP (b, red) anti-MCT2 (c, green) antibodies. TOPRO-3 was used as nuclear stain (blue). (d) Schematic representation of the hypothalamic area shown in e. (e) High magnification analysis using quadruple labeling. MCT2 labeling was negative in β1v-tanycytes (positive for vimentin) or β1d-tanycytes (positive for GFAP) or astrocytes (positive for GFAP). The MCT2 reaction was concentrated in membranes of arcuate neurons (e, arrows), and neuronal processes (e, head arrows). AN: arcuate nucleus, III–V: third ventricle, ME: median eminence, n: neurons. Scale bar: 10 µm.

In the hypothalamus, MCT2 localization was analyzed using two tanycytes markers: vimentin and GLUT1 (Fig. 7b and d). Intense MCT2 reactivity was detected in the peri-ventricular and laterals AN regions (Fig. 7c and d, arrows), excluding the cellular bodies of tanycytes (schematic representation, Fig. 7a) that form the ventricular wall (Fig. 7c, arrowheads). A positive reaction was also detected in astrocytes of the median eminence and in the terminal processes of β1d tanycytes negative for vimentin (Fig. 7c–d, asterisks). Using high magnification images, MCT2 hypothalamic cellular localization was defined (Fig. 7e–p). In the dorsal area of the peri-ventricular AN, MCT2 was detected in the hypothalamic parenchyma surrounding neuronal bodies (Fig. 7e, arrows), and co-localization with vimentin or GLUT1 was not observed (Fig. 7f–h, arrows). Co-distribution of vimentin and GLUT1 was detected only within the cellular bodies of α-tanycytes (Fig. 7h, triple arrow). In the AN ventral area, MCT2 immunoreactivity was concentrated in the peri-ventricular zone that surrounds neuronal bodies (Fig. 7i, arrows). As in the dorsal area, no co-localization between MCT2 and β1v tanycytes markers was detected (Fig. 7j–l, arrows). In the lateral AN, MCT2 formed a cellular net around the neuronal bodies (Fig. 7m, arrows), which did not express vimentin or GLUT1 (β1d tanycytes markers) (Fig. 7n–p, arrows). To corroborate the absence of co-localization between MCT2 and the glial markers, Rr values were calculated. The Rr value estimated for MCT2/vimentin was −0.012 while that for MCT2/GLUT1 was −0.027, which were significant different from the Rr value obtained for vimentin/GLUT1 control markers (0.564; *p*<0.0001), confirming the observed results. In order to corroborate MCT2 neuronal localization, the ventral peri-ventricular area of the AN was analyzed using multilabeling analysis of high magnification images (Fig. 8). β1v tanycyte projections were detected using anti-vimentin (Fig. 8a, arrows) and anti-GFAP (Fig. 8b, arrows). MCT2 was located in the hypothalamic parenchyma associated with cellular bodies and processes (Fig. 8c, arrows). No co-localization of MCT2, vimentin, and GFAP was observed, corroborating MCT2 neuronal localization in both neuronal soma (Fig. 8e, arrows) and processes (Fig. 8e, arrow heads). The Rr value estimated for MCT2/Vimentin co-localization was −0.076 and that for MCT2/GFAP co-localization was −0.040, which was significantly different from the value observed for the glial vimetin/GFAP control markers (0.192, *p*<0.0002).

### MCT2 is Localized in Orexigenic and Anorexigenic Arcuate Nucleus Neurons

**Figure 9:**
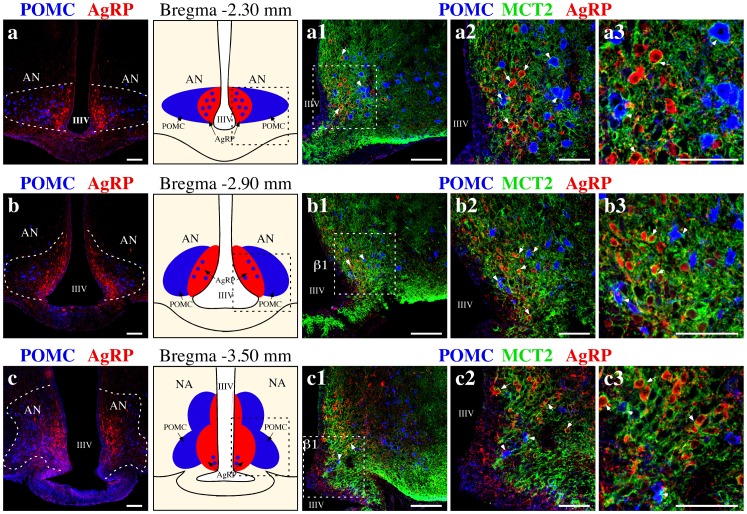
MCT2 is localized in both AgRP and POMC neurons of the arcuate nucleus. (a–c) Anteroposterior reconstruction of the basal hypothalamus. MCT2 (green), AgRP (red), POMC (blue). In the anterior region of AN (bregma −2.30 mm), MCT2 is localized in POMC neurons (a1–a3, arrowheads) and AgRP neurons (a1–a3, arrows). In the medial region of the AN (bregma −2.90 mm), MCT2 is localized mainly in AgRP neurons (b1–b3, arrows) and some POMC neurons, which are next to III–V (b1–b3, arrowheads). In the posterior region of the AN, MCT2 is segregated to AgRP neurons (arrows c1–c3) and only a few POMC neurons (c1–c3, arrowheads). AN: arcuate nucleus, IIIV: third ventricle. Scale bars: (a–c and a1–c1) 150 µm; (a2, a3, b2, b3, c2 and c3) 50 µm.

MCT2 distribution in orexigenic and anorexigenic AN neurons was evaluated in rostro-caudal rat brain slices, treated with colchicine, using anti-POMC and anti-AgRP antibodies (Fig. S2). POMC neurons are generally located distal to the third ventricle with AgRP neurons distributed proximal to ventricle, dividing AN into anorexigenic (lateral) and orexigenic (ventricular) areas (Fig. S2b–f). However, the rostral AN sub-population of POMC neurons are close to the third ventricle (Fig. S2b–c), generating a sub-compartment that contains both anorexigenic and orexigenic neurons. This neuronal distribution is reduced towards the caudal area; in the AN medial area, few POMC neurons are found within the same area as AgRP neurons (Fig. S2d). Consistently, in the caudal zone of the AN, these two populations are completely segregated (Fig. S2e–f). As shown in Figure 9, AgRP and POMC localization was analyzed in the rostral (Fig. 9a), medial (Fig. 9b) and caudal (Fig. 9c) areas. MCT2 was principally located proximal to the third ventricle surrounding primary AgRP neurons (Fig. 9a1–c1, arrows) and to a lesser degree POMC neurons (Fig. 9a1–c1, arrow heads). MCT2 was localized in neuronal membranes, and its localization was not affected by colchicine treatment (data not shown). In a more detailed analysis, changes in the localization of MCT2 from the rostral to caudal region of hypothalamus were detected. In the rostral area, MCT2-positive neurons were located in a similar proportion to AgRP and POMC neurons (95 v/s 85%) (Fig. 9a2–a3, arrows). On the contrary, in the medial AN, more AgRP than POMC neurons positive for MCT2 were observed (90 v/s 70%) (Fig. 9b2–b3, arrowheads). In the caudal zone of the AN, there were less MCT2-reactive POMC neurons, contrary to that observed for AgRP (Fig. 9c2–c3, arrows) (50 v/s 81%). Therefore, these analyses confirm the localization of MCT2 in neurons that control feeding behavior.

**Figure 10:**
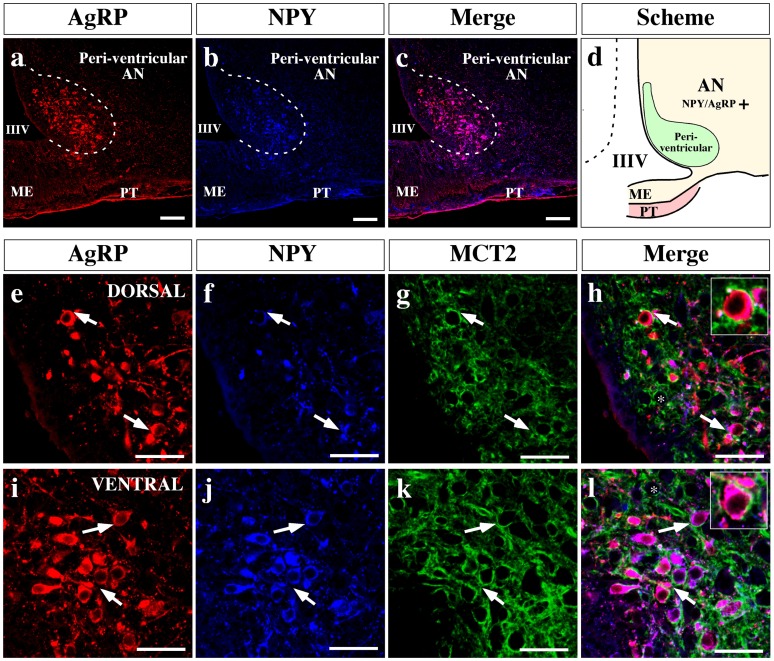
MCT2 is localized in orexigenic neurons of the arcuate nucleus. (a–c) Low magnification images of the ventro-medial hypothalamus, analyzed by immunohistochemistry using anti-AgRP (red) and anti-NPY (blue) antibodies. Ventricular arcuate nucleus neurons co-express AgRP and NPY (c). (d) Scheme summarizing the localization of the orexigenic neuropeptides in the AN. (e–l) High magnification images showing the dorsal (e–h) and ventral (i–l) region of the ventricular AN. An intense MCT2 immunoreaction was detected in orexigenic neuronal bodies in both areas (insets h and l). AN: arcuate nucleus, IIIV: third ventricle, ME: median eminence, PT: *pars tuberalis*. Scale bars: (a–c) 150 µm; (e–l) 50 µm.

**Figure 11:**
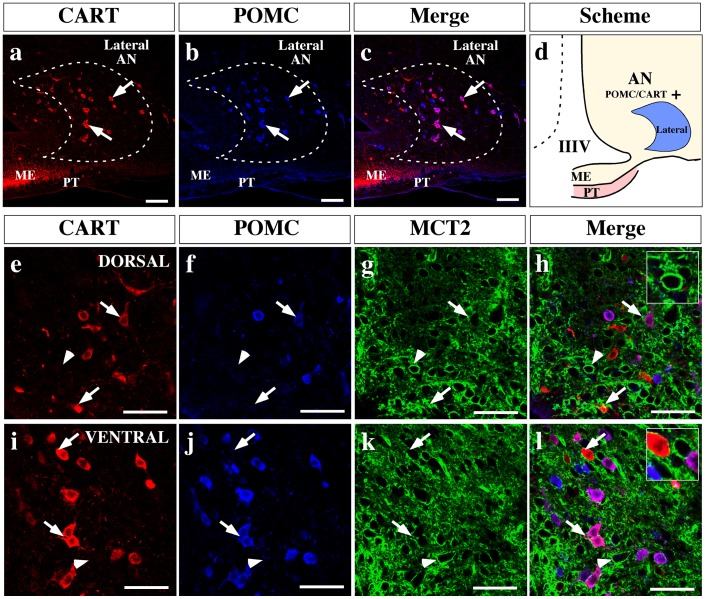
MCT2 is localized in anorexigenic neurons of AN. (a–c) Low magnification images of ventro-medial hypothalamus, analyzed by immunohistochemistry using anti-CART (red) and anti-POMC (blue) antibodies. Lateral arcuate nucleus neurons show a segregated distribution of both neuropeptides (c). (d) Scheme summarizing the localization of the anorexigenic neuropeptides in the AN. (e–l) High magnification images showing a dorsal (e–h) and ventral (i–l) region of ventricular AN. MCT2 immunoreaction was detected in some neuronal bodies that express one or both anorexigenic neuropeptides, in both areas (h and l). AN: arcuate nucleus, IIIV: third ventricle, ME: median eminence, PT: *pars tuberalis*. Scale bars: (a–c) 150 µm; (e–l) 50 µm.

Because NPY and CART neurons also participate in controlling feeding behavior and are also present in the AN, MCT2 distribution in these neurons was assessed using anti-NPY and anti-AgRP (Fig. 10) or anti- POMC and anti-CART (Fig. 11) antibodies in the AN medial area. Analyses of orexigenic markers showed that a high number of neurons located in the peri-ventricular AN co-express NPY and AgRP neuropeptides (Fig. 10a–d). High MCT2 reactivity was found surrounding orexigenic neurons in both the dorsal (Fig. 10e–h, arrows and inset) and ventral area of the peri-ventricular AN (Fig. 10i–l, arrows and inset). MCT2 distribution using anorexigenic markers in the same area was also undertaken (Fig. 11a–d). High magnification of the AN dorsal area revealed a low number of neurons that co-express POMC and CART neuropeptides (Fig. 11e–h, arrows), which is contrary to that observed in ventral area where a major number of neurons co-express both neuropeptides (Fig. 11i–l, arrows). However, MCT2 was localized in dorsal (Fig. 11e–h, arrows) and ventral (Fig. 11i–l, arrows) neurons. Furthermore, intense MCT2 expression was observed in another neuronal type that was not reactive to POMC or CART antibodies (Fig. 11h–l inset) or AgRP/NPY (Fig. 10, asterisk), suggesting that other neuronal type expressed this transporter.

## Discussion

Several neuronal populations capable of responding to changes in glucose concentration are located within the AN. These neurons couple the fluctuations in blood glucose levels with a complex network of neurochemical and neurophysiologic responses that control food intake and feeding behavior [3]. However, the mechanism by which these neurons detect changes in glucose levels it not clear. Several studies have proposed that the glucose sensing mechanism is driven by metabolic interactions between glial and neural cells that are mediated by lactate and MCTs [11], [12], [14], [15], [18]. MCT2 expression was demonstrated in the neurons of the cortex, hippocampus and cerebellum [19], [20], [33], but its function has yet to be analyzed in these cells. Because the expression and function of MCTs in hypothalamic neurons has not been determined, this study analyzed uptake of lactate through MCTs in AN neurons, showing that MCT2 is localized in neurons that control food intake.

Expression of functional MCTs was analyzed in hypothalamic neuroendocrine neurons, a mixed primary cell culture, and an immortalized hypothalamic cell line (GT1-7) in the present study. In primary cultures, MCT2 was expressed in neuroendocrine neurons; however, limitations of the fluorimetric assays prevented further functional characterization of the MCTs expressed in these cells. Lactate uptake was observed in hypothalamic neurons *in vivo*. Because neurons cultured *in vitro* induced the expression of MCT1 and MCT4, transporters with high transport capacity (Km 6 and 55 mM, respectively) [34], [35], their expression may mask MCT2 (Km 0.5 mM) [21], [36]. Therefore, the kinetic characterization of MCT2 was performed in GT1-7 cells using radiometric methods. RT-PCR and Western blot analyses confirmed the expression of MCT1 and MCT2 in these cells, demonstrating that this immortalized cell culture is a suitable model of neuroendocrine neurons to evaluate MCT function. Transport and competition assays revealed that GT1-7 cells express functionally active MCT1 and MCT2. The lower affinity transport component had the expected properties of MCT1, with a transport Km of 6 mM [34], [37]. In contrast, the higher affinity transporter revealed an apparent Km of approximately 0.5 mM, which fits the description of MCT2 [21], [36]. Furthermore, increased lactate uptake was observed with lower pH values, which is similar to that previously reported for MCT1 and MCT2 [21], [34], [36], [37]. Lactate transport was strongly inhibited by 10 mM pyruvate as both isoforms transport this substrate [21], [34], [37], [38]. Additionally, 1 mM floretin decreased lactate uptake by 70%, according with the IC_50_ reported for MCT1 (30 µM) and MCT2 (15 µM) [21], [34], [37]. 4-CIN also strongly inhibited lactate uptake, which is in accordance with previous findings [21], [34], [37]). In addition, a smaller but significant inhibitory effect on lactate uptake was observed with 1 mM DIDS, which was similar to that observed in MCT-expressing oocytes [21], [37]. However, 3 mM pCMBS, an inhibitor of MCT1 but not MCT2 [21], [34], did not significantly inhibit lactate uptake, indicating that MCT2 is the preferred transporter for lactate incorporation in GT1-7 neuronal cells.

The *in vitro* expression and functional analyses of the present study indicated that MCT2 is used to incorporate lactate in the hypothalamic neurons. However, only one study demonstrated *in situ* MCT2 expression in the hypothalamic neurons of rats fed a high fat diet, which induced MCT2 over-expression in several hypothalamic nuclei including the AN [39]. The present study is the first to demonstrate MCT2 expression in hypothalamic AN neuroendocrine neurons in rats, under normal dietary conditions, supporting the idea of a glucose sensing mechanism driven by glia-neuron interactions through lactate.

RT-PCR and immunoblot analyses revealed that MCT2 is expressed in hypothalamic tissue, which was confirmed by *in situ* hybridization and immunohistochemical analyses. Both studies showed high MCT2 expression in hypothalamic neurons localized in the peri-ventricular and lateral regions of AN. However, several neuronal populations with antagonistic actions on the food intake, namely orexigenic and anorexigenic neurons, are localized within the hypothalamus [4]. In the present study, detailed immunohistochemical analyses showed that MCT2 is localized in peri-ventricular and distal AN neurons in both cellular populations. However, this distribution depends on the hypothalamic area examined. For example, a similar number of orexigenic and anorexigenic neurons reactive to MCT2 were observed in the rostral area of the basal hypothalamus. In contrast, in the more caudal areas, MCT2 was primarily localized within NPY and AgRP neurons. This change in MCT2 distribution in orexigenic and anorexigenic AN neurons is correlated with the localization of glucose-inhibited (GI) and glucose-exited (GE) neurons [40]. NPY neurons are GI neurons, which reduce their electrical activity in response to high glucose [7], but the electrical-glucose identity of anorexigenic neurons (POMC and CART) has not been completely elucidated [8], [9], [41]. Electrophysiology studies performed in transgenic mice indicate that these neurons could be corresponding to GE neurons [8], [9]. Thus, neuroendocrine neurons may couple the neuronal activity with changes in glucose concentration and feeding behavior. Therefore, AN may represent an anteroposterior compartment in which MCT2 localization generates metabolic niches for orexigenic (GI) and anorexigenic (GE) neurons, controlling food intake and feeding behavior (discussed later).

Several studies support the idea that lactate is a key signal in hypothalamic glucose sensing and food intake [18], [42], [43], [44], [45]. Furthermore, lactate perfusion of the VMH suppresses the hypoglycemic counter-regulatory response, with a strong diminution in glucagon and epinephrine release [42]. Moreover, central inhibition of lactate dehydrogenase by administration of oxamate abolishes the effects of ICV-glucose on blood glucose lowering. Thus, blocking lactate metabolism in the hypothalamus results in a 40% reduction in the inhibitory action of glucose on circulating glucose levels [18]. Besides, ICV injection of lactate reduces blood glucose levels and food intake, resulting in body weight loss in eight-week-old rats [45]. Interestingly, the inhibition of lactic dehydrogenase or K_ATP_ channels in the hypothalamus increases glucose blood levels in the presence of systemic lactate [43]. These findings provide evidence for both a direct intracellular mechanism of K_ATP_ channel regulation, which does not involve increased [ATP]_c_ [46], as well as a separate intercellular signaling mechanism mediated by lactate released from neighboring glial cells.

Based on the results presented here and our previous studies, we postulate that neuron-glial interactions through lactate regulate the activity of neurons that control food intake and feeding behavior. The specific localization of tanycytes in direct contact with cerebrospinal fluid [10] coupled with GLUT2/GK expression strongly [12], [15] supports the idea that these cells have a high capacity to uptake glucose. Furthermore, our recent work has shown that tanycytes have the capability to increase intracellular calcium levels in response to high glucose [16], demonstrating that these cells can respond to glucose. Furthermore, tanycytes can release lactate through both MCT1 and MCT4 [11]. The coincident expression of MCT2 in orexigenic neurons with the localization MCT1 in β1v-tanycytes distributed in the peri-ventricular area [11] led us to propose that these glial cells regulate the activity of GI anorexigenic neurons. We postulate that lactate released by tanycytes, but not glucose itself; reduces the firing rate of these neurons in hyperglycemic conditions. Moreover, the localization of MCT4 in β1d-tanycytes suggests that these cells could couple with anorexigenic neurons located in the lateral AN through MCT2. In high glucose, these neurons uptake the lactate released by tanycytes, increasing their firing rate and generating satiety through two feasible pathways. The first pathway is mediated by anorexigenic neuropeptides, including α-MSH (POMC-derived), from GE-anorexigenic neurons [8], [9]. The second alternative is an indirect pathway where GE and anorexigenic neurons are two different cells [41], and therefore the GE neuron regulates the secretion of anorexigenic neuropeptides.

In conclusion, we postulated that lactate has a dual role in the control of the feeding behavior, depending on the subtype of neuronal and glial cells activated in the process. This evidence suggests an important rol for tanycytes in the glucosensing mechanism driven by lactate for indirectly controlling the neuronal function.

## Supporting Information

Figure S1 MCT2 is localized in kidney and brain tissue. (a) Immunohistochemical localization of MCT2 in the basolateral region of the collecting tubules of the renal medulla (arrows). (b) MCT2 is expressed in capillaries in the cerebral cortex (arrows). (c–h) Co-distribution of MCT2 (green) and GLUT1 (red) in the CNS. MCT2 is localized in astrocytes surrounding blood vessels positive for GLUT1 (c-d, arrows), which form the marginal glia (e, arrows); co-distribution was only detected in the microvasculature (c–d, arrow heads). An intense MCT2 reaction was detected in the lateral ventricle ependymal cells (f-g, arrows) and subependymal astrocytes (h, arrows). Absence of co-localization with GLUT1 was observed in the areas analyzed. CD: collecting ducts, CP: choroid plexus, LV: lateral ventricle, IIIV: third ventricle. Scale bar: 50 µm.
(JPG)

Figure S2 POMC and AgRP rostro-caudal distribution. (a) Schematic representation of rat brain showing the sections analyzed. (b–f) Immunolocalization using anti-POMC (blue) and anti-AgRP (red) antibodies. Scale bar: 150 µm.
(TIF)

## References

[1] Oomura Y, Yoshimatsu H (1984) Neural network of glucose monitoring system. J Auton Nerv Syst 10: 359–372. 6090526 10.1016/0165-1838(84)90033-x

[2] Yang XJ, Kow LM, Funabashi T, Mobbs CV (1999) Hypothalamic glucose sensor: similarities to and differences from pancreatic beta-cell mechanisms. Diabetes 48: 1763–1772. 10480606 10.2337/diabetes.48.9.1763

[3] Blouet C, Schwartz GJ (2010) Hypothalamic nutrient sensing in the control of energy homeostasis. Behav Brain Res 209: 1–12. 20035790 10.1016/j.bbr.2009.12.024

[4] Schwartz MW, Woods SC, Porte D Jr, Seeley RJ, Baskin DG (2000) Central nervous system control of food intake. Nature 404: 661–671. 10766253 10.1038/35007534

[5] Levin BE, Routh VH, Kang L, Sanders NM, Dunn-Meynell AA (2004) Neuronal glucosensing: what do we know after 50 years? Diabetes 53: 2521–2528. 15448079 10.2337/diabetes.53.10.2521

[6] Song Z, Routh VH (2005) Differential effects of glucose and lactate on glucosensing neurons in the ventromedial hypothalamic nucleus. Diabetes 54: 15–22. 15616006 10.2337/diabetes.54.1.15

[7] Muroya S, Yada T, Shioda S, Takigawa M (1999) Glucose-sensitive neurons in the rat arcuate nucleus contain neuropeptide Y. Neurosci Lett. 264: 113–116. 10.1016/s0304-3940(99)00185-8 10320027

[8] Ibrahim N, Bosch MA, Smart JL, Qiu J, Rubinstein M, et al (2003) Hypothalamic proopiomelanocortin neurons are glucose responsive and express K(ATP) channels. Endocrinology 144: 1331–1340. 12639916 10.1210/en.2002-221033

[9] Parton LE, Ye CP, Coppari R, Enriori PJ, Choi B, et al (2007) Glucose sensing by POMC neurons regulates glucose homeostasis and is impaired in obesity. Nature 449: 228–232. 17728716 10.1038/nature06098

[10] Akmayev IG, Popov AP (1977) Morphological aspects of the hypothalamic-hypophyseal system. VII. The tanycytes: Their relation to the hypophyseal adrenocorticotrophic function. An ultrastructural study. Cell Tissue Res 180: 263–282. 194701 10.1007/BF00231958

[11] Cortes-Campos C, Elizondo R, Llanos P, Uranga RM, Nualart F, et al (2011) MCT expression and lactate influx/efflux in tanycytes involved in glia-neuron metabolic interaction. PLoS One 6: e16411. 21297988 10.1371/journal.pone.0016411 PMC3030577

[12] Garcia MA, Millan C, Balmaceda-Aguilera C, Castro T, Pastor P, et al (2003) Hypothalamic ependymal-glial cells express the glucose transporter GLUT2, a protein involved in glucose sensing. J Neurochem 86: 709–724. 12859684 10.1046/j.1471-4159.2003.01892.x

[13] Rodriguez EM, Blazquez JL, Pastor FE, Pelaez B, Pena P, et al (2005) Hypothalamic tanycytes: a key component of brain-endocrine interaction. Int Rev Cytol 247: 89–164. 16344112 10.1016/S0074-7696(05)47003-5

[14] Ainscow EK, Mirshamsi S, Tang T, Ashford ML, Rutter GA (2002) Dynamic imaging of free cytosolic ATP concentration during fuel sensing by rat hypothalamic neurones: evidence for ATP-independent control of ATP-sensitive K(+) channels. J Physiol 544: 429–445. 12381816 10.1113/jphysiol.2002.022434 PMC2290605

[15] Millan C, Martinez F, Cortes-Campos C, Lizama I, Yanez MJ, et al (2010) Glial glucokinase expression in adult and post-natal development of the hypothalamic region. ASN Neuro 2: e00035. 20531973 10.1042/AN20090059 PMC2881537

[16] Orellana JA, Saez PJ, Cortes-Campos C, Elizondo RJ, Shoji KF, et al (2012) Glucose increases intracellular free Ca(2+) in tanycytes via ATP released through connexin 43 hemichannels. Glia 60: 53–68. 21987367 10.1002/glia.21246 PMC3417330

[17] Thorens B (2012) Sensing of glucose in the brain. Handb Exp Pharmacol: 277–294. 10.1007/978-3-642-24716-3_12 22249819

[18] Lam TK, Gutierrez-Juarez R, Pocai A, Rossetti L (2005) Regulation of blood glucose by hypothalamic pyruvate metabolism. Science 309: 943–947. 16081739 10.1126/science.1112085

[19] Bergersen L, Waerhaug O, Helm J, Thomas M, Laake P, et al (2001) A novel postsynaptic density protein: the monocarboxylate transporter MCT2 is co-localized with delta-glutamate receptors in postsynaptic densities of parallel fiber-Purkinje cell synapses. Exp Brain Res 136: 523–534. 11291733 10.1007/s002210000600

[20] Bergersen LH, Magistretti PJ, Pellerin L (2004) Selective Postsynaptic Co-localization of MCT2 with AMPA Receptor GluR2/3 Subunits at Excitatory Synapses Exhibiting AMPA Receptor Trafficking. Cereb Cortex. 10.1093/cercor/bhh138 15749979

[21] Broer S, Broer A, Schneider HP, Stegen C, Halestrap AP, et al (1999) Characterization of the high-affinity monocarboxylate transporter MCT2 in Xenopus laevis oocytes. Biochem J 341 (Pt 3): 529–535. 10.1042/0264-6021:3410529 PMC1220388 10417314

[22] Pierre K, Magistretti PJ, Pellerin L (2002) MCT2 is a major neuronal monocarboxylate transporter in the adult mouse brain. J Cereb Blood Flow Metab 22: 586–595. 11973431 10.1097/00004647-200205000-00010

[23] Pierre K, Pellerin L, Debernardi R, Riederer BM, Magistretti PJ (2000) Cell-specific localization of monocarboxylate transporters, MCT1 and MCT2, in the adult mouse brain revealed by double immunohistochemical labeling and confocal microscopy. Neuroscience 100: 617–627. 11098125 10.1016/s0306-4522(00)00294-3

[24] Mellon PL, Windle JJ, Goldsmith PC, Padula CA, Roberts JL, et al (1990) Immortalization of hypothalamic GnRH neurons by genetically targeted tumorigenesis. Neuron 5: 1–10. 2196069 10.1016/0896-6273(90)90028-e

[25] Chesler M (2003) Regulation and modulation of pH in the brain. Physiol Rev 83: 1183–1221. 14506304 10.1152/physrev.00010.2003

[26] Garcia Mde L, Salazar K, Millan C, Rodriguez F, Montecinos H, et al (2005) Sodium vitamin C cotransporter SVCT2 is expressed in hypothalamic glial cells. Glia 50: 32–47. 15625716 10.1002/glia.20133

[27] Manders EM, Stap J, Brakenhoff GJ, van Driel R, Aten JA (1992) Dynamics of three-dimensional replication patterns during the S-phase, analysed by double labelling of DNA and confocal microscopy. J Cell Sci 103 (Pt 3): 857–862. 10.1242/jcs.103.3.857 1478975

[28] Liposits Z, Merchenthaler I, Wetsel WC, Reid JJ, Mellon PL, et al (1991) Morphological characterization of immortalized hypothalamic neurons synthesizing luteinizing hormone-releasing hormone. Endocrinology 129: 1575–1583. 1874189 10.1210/endo-129-3-1575

[29] Eladari D, Chambrey R, Irinopoulou T, Leviel F, Pezy F, et al (1999) Polarized expression of different monocarboxylate transporters in rat medullary thick limbs of Henle. J Biol Chem 274: 28420–28426. 10497203 10.1074/jbc.274.40.28420

[30] Garcia CK, Brown MS, Pathak RK, Goldstein JL (1995) cDNA cloning of MCT2, a second monocarboxylate transporter expressed in different cells than MCT1. J Biol Chem 270: 1843–1849. 7829520 10.1074/jbc.270.4.1843

[31] Gerhart DZ, Enerson BE, Zhdankina OY, Leino RL, Drewes LR (1998) Expression of the monocarboxylate transporter MCT2 by rat brain glia. Glia 22: 272–281. 9482213

[32] Hanu R, McKenna M, O'Neill A, Resneck WG, Bloch RJ (2000) Monocarboxylic acid transporters, MCT1 and MCT2, in cortical astrocytes in vitro and in vivo. Am J Physiol Cell Physiol 278: C921–930. 10794666 10.1152/ajpcell.2000.278.5.C921

[33] Pierre K, Pellerin L (2005) Monocarboxylate transporters in the central nervous system: distribution, regulation and function. J Neurochem 94: 1–14. 10.1111/j.1471-4159.2005.03168.x 15953344

[34] Broer S, Rahman B, Pellegri G, Pellerin L, Martin JL, et al (1997) Comparison of lactate transport in astroglial cells and monocarboxylate transporter 1 (MCT 1) expressing Xenopus laevis oocytes. Expression of two different monocarboxylate transporters in astroglial cells and neurons. J Biol Chem 272: 30096–30102. 9374487 10.1074/jbc.272.48.30096

[35] Dimmer KS, Friedrich B, Lang F, Deitmer JW, Broer S (2000) The low-affinity monocarboxylate transporter MCT4 is adapted to the export of lactate in highly glycolytic cells. Biochem J 350 Pt 1: 219–227. PMC1221245 10926847

[36] Lin RY, Vera JC, Chaganti RS, Golde DW (1998) Human monocarboxylate transporter 2 (MCT2) is a high affinity pyruvate transporter. J Biol Chem 273: 28959–28965. 9786900 10.1074/jbc.273.44.28959

[37] Broer S, Schneider HP, Broer A, Rahman B, Hamprecht B, et al (1998) Characterization of the monocarboxylate transporter 1 expressed in Xenopus laevis oocytes by changes in cytosolic pH. Biochem J 333 (Pt 1): 167–174. 10.1042/bj3330167 PMC1219569 9639576

[38] Garcia CK, Goldstein JL, Pathak RK, Anderson RG, Brown MS (1994) Molecular characterization of a membrane transporter for lactate, pyruvate, and other monocarboxylates: implications for the Cori cycle. Cell 76: 865–873. 8124722 10.1016/0092-8674(94)90361-1

[39] Pierre K, Parent A, Jayet PY, Halestrap AP, Scherrer U, et al (2007) Enhanced expression of three monocarboxylate transporter isoforms in the brain of obese mice. J Physiol 583: 469–486. 17599960 10.1113/jphysiol.2007.138594 PMC2277016

[40] Wang R, Liu X, Hentges ST, Dunn-Meynell AA, Levin BE, et al (2004) The regulation of glucose-excited neurons in the hypothalamic arcuate nucleus by glucose and feeding-relevant peptides. Diabetes 53: 1959–1965. 15277373 10.2337/diabetes.53.8.1959

[41] Fioramonti X, Contie S, Song Z, Routh VH, Lorsignol A, et al (2007) Characterization of glucosensing neuron subpopulations in the arcuate nucleus: integration in neuropeptide Y and pro-opio melanocortin networks? Diabetes 56: 1219–1227. 17261674 10.2337/db06-0567

[42] Borg MA, Tamborlane WV, Shulman GI, Sherwin RS (2003) Local lactate perfusion of the ventromedial hypothalamus suppresses hypoglycemic counterregulation. Diabetes 52: 663–666. 12606506 10.2337/diabetes.52.3.663

[43] Kokorovic A, Cheung GW, Rossetti L, Lam TK (2009) Hypothalamic sensing of circulating lactate regulates glucose production. J Cell Mol Med 13: 4403–4408. 19040414 10.1111/j.1582-4934.2008.00596.x PMC4515055

[44] Lam CK, Chari M, Lam TK (2009) CNS regulation of glucose homeostasis. Physiology (Bethesda) 24: 159–170. 19509126 10.1152/physiol.00003.2009

[45] Lam CK, Chari M, Wang PY, Lam TK (2008) Central lactate metabolism regulates food intake. Am J Physiol Endocrinol Metab 295: E491–496. 18577696 10.1152/ajpendo.90481.2008

[46] Gonzalez JA, Reimann F, Burdakov D (2009) Dissociation between sensing and metabolism of glucose in sugar sensing neurones. J Physiol 587: 41–48. 18981030 10.1113/jphysiol.2008.163410 PMC2670021

