# Supplementary figures and images for: MCT2 Expression and Lactate Influx in Anorexigenic and Orexigenic Neurons of the Arcuate Nucleus
Source: PLoS One. 2013 Apr 26;8(4):e62532. doi: 10.1371/journal.pone.0062532 (PMC3637215; doi:10.1371/journal.pone.0062532)

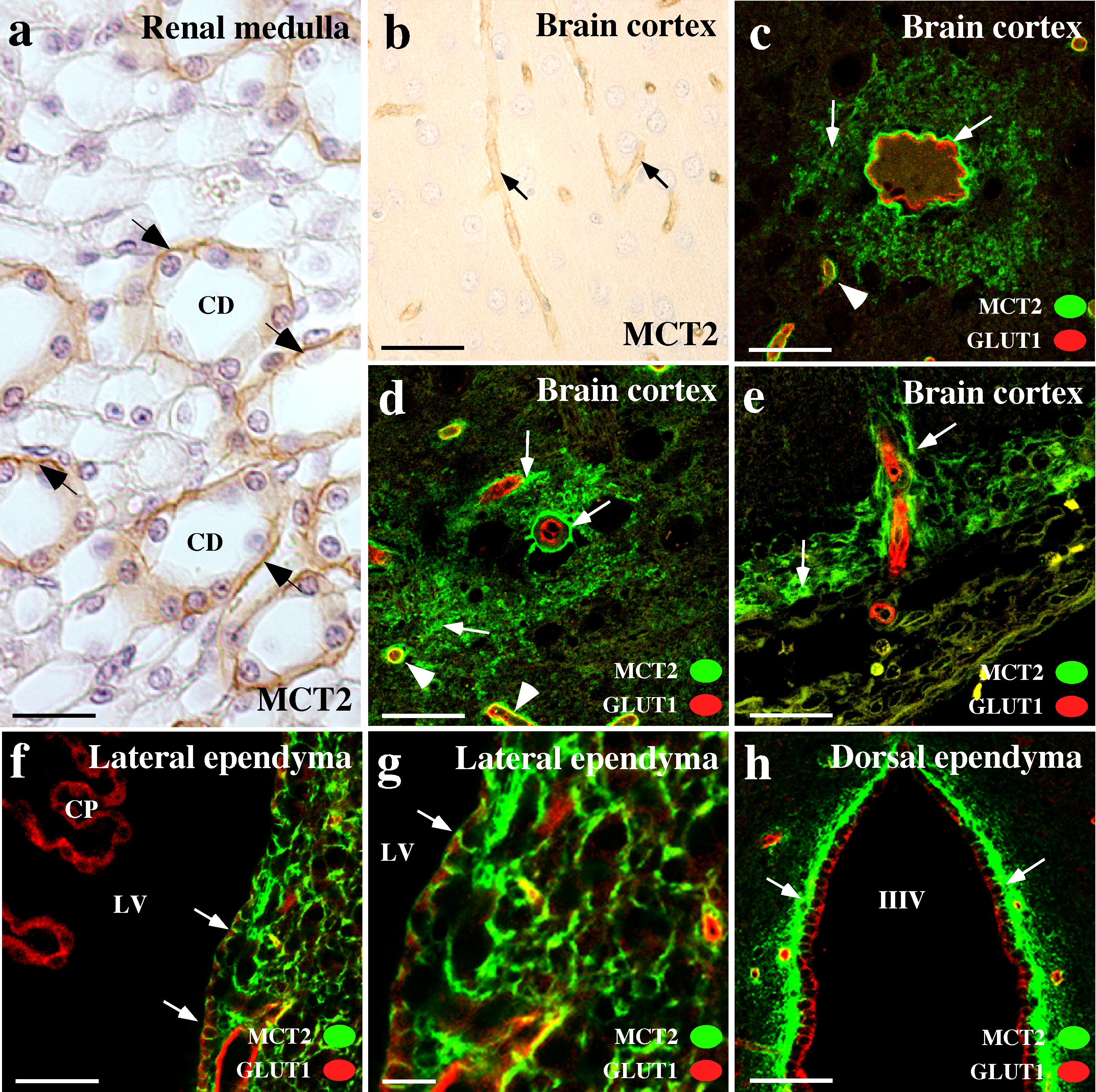

Supplement: Figure S1 — MCT2 is localized in kidney and brain tissue. (a) Immunohistochemical localization of MCT2 in the basolateral region of the collecting tubules of the renal medulla (arrows). (b) MCT2 is expressed in capillaries in the cerebral cortex (arrows). (c–h) Co-distribution of MCT2 (green) and GLUT1 (red) in the CNS. MCT2 is localized in astrocytes surrounding blood vessels positive for GLUT1 (c-d, arrows), which form the marginal glia (e, arrows); co-distribution was only detected in the microvasculature (c–d, arrow heads). An intense MCT2 reaction was detected in the lateral ventricle ependymal cells (f-g, arrows) and subependymal astrocytes (h, arrows). Absence of co-localization with GLUT1 was observed in the areas analyzed. CD: collecting ducts, CP: choroid plexus, LV: lateral ventricle, IIIV: third ventricle. Scale bar: 50 µm. (JPG) [file pone.0062532.s001.jpg]

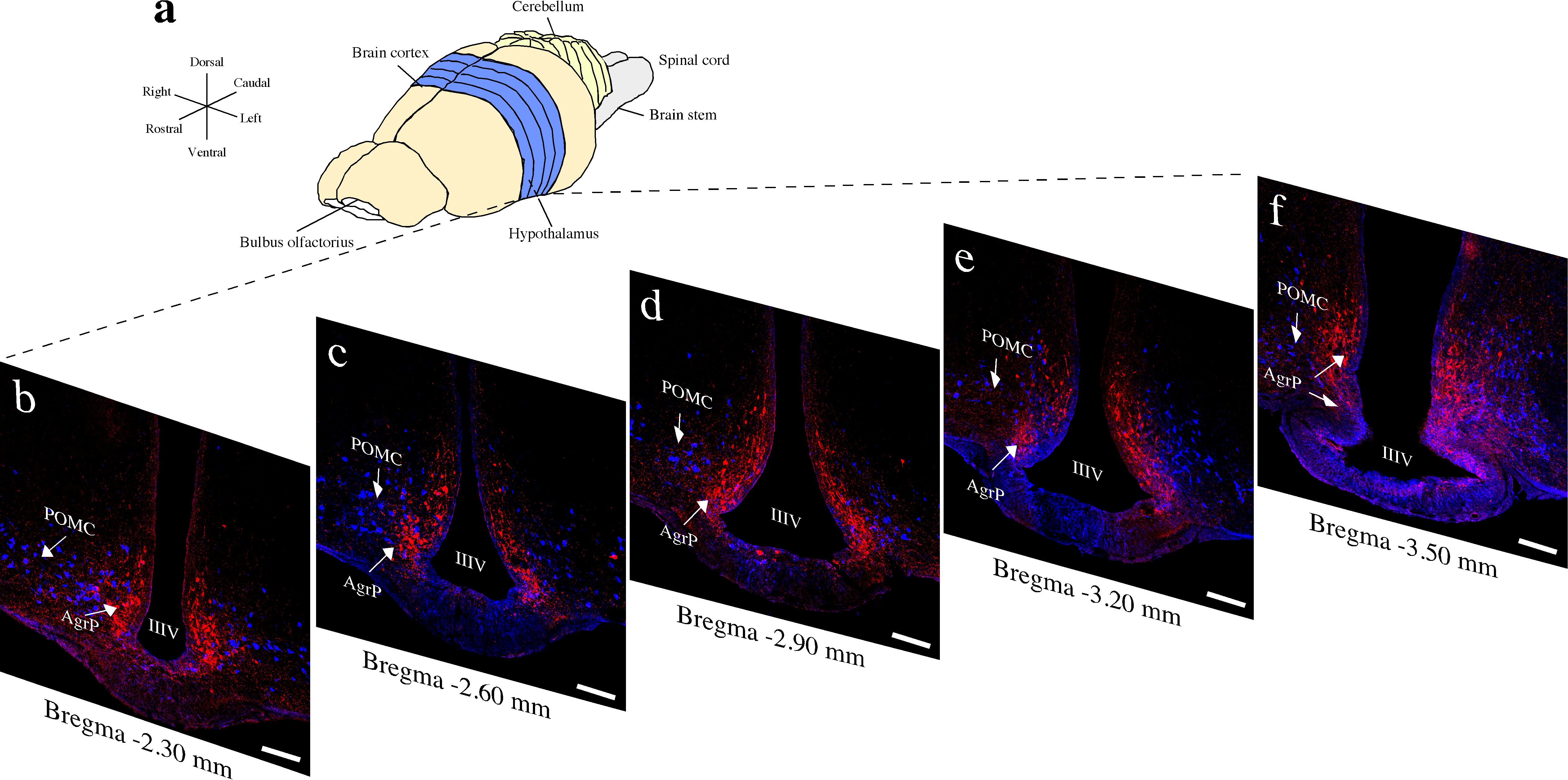

Supplement: Figure S2 — POMC and AgRP rostro-caudal distribution. (a) Schematic representation of rat brain showing the sections analyzed. (b–f) Immunolocalization using anti-POMC (blue) and anti-AgRP (red) antibodies. Scale bar: 150 µm. (TIF) [file pone.0062532.s002.tif]
